# An association between visceral or subcutaneous fat accumulation and diabetes mellitus among Japanese subjects

**DOI:** 10.1186/s13098-021-00646-3

**Published:** 2021-04-14

**Authors:** Hirohide Yokokawa, Hiroshi Fukuda, Mizue Saita, Kento Goto, Tengen Kaku, Taiju Miyagami, Yuichi Takahashi, Chieko Hamada, Teruhiko Hisaoka, Toshio Naito

**Affiliations:** 1 Department of General Medicine, Juntendo University Faculty of Medicine, Hongo 2-1-1, Bunkyo-ku, Tokyo, 113-8421 Japan; 2 Department of General Medicine, Juntendo University School of Medicine, Hongo 2-1-1, Bunkyo-ku, Tokyo, 113-8421 Japan

**Keywords:** Lifestyle‐related disorder, Prevention, Epidemiology, Subcutaneous fat, Visceral fat, Diabetes mellitus, Metabolic syndrome

## Abstract

**Background:**

The impact of subcutaneous fat accumulation remains controversial. This study assessed the association between visceral or subcutaneous fat area (VFA and SFA, respectively) and diabetes mellitus (DM) among Japanese subjects.

**Methods:**

This was a cross-sectional study involving 1907 eligible participants (men, 1050; women, 857) who participated in a voluntary health check-up conducted at Juntendo University Hospital from January 2017 to December 2018, in Tokyo, Japan. Associations between VFA or SFA quartiles and DM were identified using adjusted odds ratios (AORs) and 95% confidence intervals (CIs) with multivariable logistic regression analysis adjusted for confounders. Receiver operating characteristic (ROC) curve analysis was used to assess appropriate cut-off values of VFA or SFA.

**Results:**

Multivariate analyses showed that Q4 (≥ 125 cm^2^) of VFA was significantly positively associated with DM compared to Q1 (< 65 cm^2^) (AOR = 1.94, 95% CI 1.02–3.71), whereas there was no association between SFA and DM in men. Among women, Q4 (≥ 85 cm^2^) of VFA was significantly positively associated with DM compared to Q1 (< 30 cm^2^) (Q4, AOR = 6.15, 95% CI 1.65–22.99). Also, Q3 and Q4 (≥ 135 cm^2^) of SFA were significantly positively associated with DM compared to Q1 (< 90 cm^2^) (Q3, AOR = 5.64, 95% CI 1.21–26.25; Q4, AOR = 7.81, 95% CI 1.71–35.65). The appropriate cut-off value of VFA in men was 101.5 cm^2^. Those of VFA and SFA in women were 72.5 cm^2^ and 165.3 cm^2^, respectively.

**Conclusions:**

Our results suggest the importance of considering SFA as well as VFA, especially in women, for primary and secondary prevention of DM.

## Introduction

Type 2 diabetes mellitus (DM) has become a highly prevalent disease worldwide as changing lifestyles have led to reduced physical activity and increased obesity, and it is now recognized as major public health burden. The prevalence of both type 2 DM and obesity has increased worldwide over the last century, not only in developed countries but also developing countries, sometimes coexisting with undernutrition [1]. It was estimated that in 2017 there were 451 million (age 18–99 years) people with diabetes worldwide. These figures are expected to increase to 693 million by 2045, and it is estimated that almost half of all people (49.7%) living with diabetes are undiagnosed. In 2017, approximately 5 million deaths worldwide in persons in the 20–99 years age range were attributed to diabetes [1].

Obesity, especially abdominal obesity, is a well-known underlying risk factor for the development of diabetes [2]. Visceral fat accumulation, which is a key feature of abdominal obesity, is in an upstream position in the pathogenesis and development of metabolic syndrome (Mets) with clustering of DM, dyslipidemia, and hypertension [2]. In particular, several epidemiologic studies have shown that excess visceral fat area (VFA) is a well-known risk factor for the development of DM and onset of cardiovascular disorders [2, 3]. Therefore, measurement of visceral fat accumulation is important to assess subjects with high risk of DM and other cardiovascular disorders [2–4]. As for VFA cut-off values for Mets, there are several differences associated with age distribution, the number of study participants, degree of obesity, and ethnicity [2–5]. In Japan, visceral fat accumulation is generally recognized as a waist circumference (WC) of over 85 cm in men and over 90 cm in women, which correspond to a VFA of 100 cm^2^ in an abdominal computed tomography (CT) scan at the umbilical level [3].

However, the impact of subcutaneous fat accumulation remains controversial. Several previous reports indicated a protective effect for glucose metabolism [6, 7]. In contrast, there is some evidence indicating a positive association between subcutaneous fat accumulation and adverse cardiometabolic risk factors, including diabetes [8, 9]. There are differences between subcutaneous adipose tissue (SCAT) and visceral adipose tissue (VAT) present in the abdominal cavity [10], including anatomic, cellular, molecular, physiologic, clinical, and prognostic differences. Anatomically, VAT is present mainly in the mesentery and omentum and drains directly through the portal circulation to the liver. Compared with SCAT, VAT is more cellular, vascular, and innervated, contains a larger number of inflammatory and immune cells, has less pre-adipocyte differentiating capacity, and has a greater percentage of large adipocytes [10].

The present study assessed the association between DM and VFA and subcutaneous fat area (SFA) estimated by CT scans in Japanese subjects.

## Subjects and methods

This was a cross-sectional study that screened 2885 Japanese adults who participated in a voluntary health checkup conducted at Juntendo University Hospital from January 2017 to December 2018, in Tokyo, Japan. A total of 978 subjects were excluded due to missing data, and 1 subject was excluded due to a duplicate case. Ultimately, 1907 participants were included in the present study (men, 1050; women, 857).

### Variables

Height, weight, body mass index (BMI), and WC were measured with participants in the standing position. BMI was calculated by dividing body weight (kg) by height squared (m^2^). Mean systolic blood pressure (SBP) and diastolic blood pressure (DBP) were calculated from the means of two upper-arm blood pressure measurements taken from participants who had been seated for at least 5 min. Serum levels of total cholesterol (mg/dl; TC), high-density-lipoprotein cholesterol (mg/dl; HDL-C), low-density-lipoprotein cholesterol (mg/dl; LDL-C), and triglycerides (mg/dl; TGs) were also measured. LDL-C was estimated using the Friedewald equation [(TC) – (HDL-C) – (TG/5)] [11]. Hemoglobin A1c (HbA1c) levels were determined by high-performance liquid chromatography using an automated analyzer. Serum uric acid (mg/dl) and high-sensitivity C-reactive protein (mg/dl) were also measured.

Participants were asked to complete a self-administered questionnaire that addressed healthy lifestyle characteristics based on Breslow’s seven health practices [12]. These characteristics can be used to assess lifestyle health, and strong associations have been found between healthy lifestyle practices and successful blood pressure control among patients with hypertension [13]. Healthy lifestyle items in the questionnaire included non-daily alcohol consumption, non-smoker status, exercise frequency of two or more times per week, BMI of 18.5–24.9 kg/m^2^, adequate sleep duration, daily breakfast consumption, and no snacking between meals [12, 13].

From the self-administered questionnaire, we also collected information on present medical history of comorbidities, such as hypertension, DM, dyslipidemia, hyperuricemia, cardiovascular disease, and cerebrovascular disease. If participants answered as having these comorbidities, we registered the participants with a medical history of these comorbidities (present).

### CT measurement of abdominal adipose tissue

Abdominal fat area, including VFA and SFA, was measured from CT scans taken at the level of the umbilicus while in the supine position and during late expiration according to Japanese guidelines for obesity treatment [14]. An Aquilion ONE/GENESIS Edition CT scanner (Canon Medical Systems Corp., Tokyo, Japan) was used to obtain CT scans. We manually traced the inner aspect of the whole trunk, muscular layer, and the abdominal wall. In the computerized method using commercial software designed for quantification of VFA and SFA (Canon Medical Systems Corp.), fat was defined as any tissue with a threshold of − 150 to − 70 Hounsfield units. Abdominal VFA was defined as the fat area enclosed by the inner aspect of the abdominal wall, and SFA was defined as the fat area enclosed by the outer aspect of the abdominal wall [15, 16]. The method is widely used and a previous study indicated that CT and magnetic-resonance imaging (MRI) may yield different absolute values of fat areas (especially visceral fat) but that the ranking of individuals on the basis of their fat areas will be similar by both methods [17, 18].

### Definition of lifestyle‐related disorders

Lifestyle-related disorders were defined according to the following criteria: (1) DM, high fasting plasma glucose (≥ 126 mg/dl) or HbA1c (≥ 6.5%) or taking antidiabetes medications; (2) hypertension, increased blood pressure (SBP ≥ 140 mmHg or DBP ≥ 90 mmHg) or taking antihypertensive medications; (3) dyslipidemia, increased TG level (≥ 150 mg/dl) or LDL-C level (≥ 140 mg/dl) or reduced HDL-C level (< 40 mg/dl) or taking dyslipidemia medications [3].

### Statistical analysis

Results are presented as mean ± standard deviation (SD) for continuous variables or prevalence (%) for categorical variables by sex. VFA and SFA quartiles were defined by sex [men; VFA (cm^2^): Q1 < 65, 65 ≤ Q2 < 95, 95 ≤ Q3 < 125, 125 ≤ Q4: SFA (cm^2^): Q1 < 85, 85 ≤ Q2 < 115, 115 ≤ Q3 < 155, 155 ≤ Q4], [women; VFA (cm^2^): Q1 < 30, 30 ≤ Q2 < 60, 60 ≤ Q3 < 85, 85 ≤ Q4: SFA (cm^2^): Q1 < 90, 85 ≤ Q2 < 135, 135 ≤ Q3 < 190, 190 ≤ Q4]. Associations between VFA or SFA quartiles and DM were identified using adjusted odds ratios (AORs) and 95% confidence intervals (CIs) with multivariable logistic regression analysis adjusted for age (years), dyslipidemia (yes), hypertension (yes), hyperuricemia (yes), alcohol consumption (non-daily drinker), and smoking (non-smoker).

Receiver operating characteristic (ROC) curve analysis was used to assess appropriate cut-off values of VFA and SFA, and we estimated the area under the curve (AUC) and measured the sensitivity and specificity for DM in both sexes. All statistical analyses were performed using the Statistical Package for Social Sciences, version 22 (SPSS Inc., Chicago, IL, USA).

The research protocol was reviewed and approved by the Ethics Committee of the Juntendo University Hospital (no. 18-297), and written informed consent was obtained from all participants.

## Results

The mean age (SD) of non-DM and DM was 59.9 (12.3) and 65.7 (8.9) years in men and 60.2 (12.9) and 66.5 (9.4) years in women, respectively (Table 1). Participants with DM had significantly higher mean BMI, WC, and VFA compared to non-DM participants. The mean SFA of DM participants was significantly higher than that of non-DM participants among women, whereas no statistically significant difference was observed among men.

**Table 1:** Sex-specific characteristics (n = 1907)

	Men (n = 1050)					Women (n = 857)				
	Mean (SD) or n (%)					Mean (SD) or n (%)				
	Non-DM (n = 868)		DM (n = 182)			Non-DM (n = 802)		DM (n = 55 )		
Age (years)	59.9 (12.3)		65.7 (8.9)		< 0.01	60.2 (12.9)		66.5 (9.4)		< 0.01
Anthropometric measurements										
Body mass index (kg/m^2^)	24.3 (3.1)		25.2 (3.6)		< 0.01	21.8 (3.4)		24.5 (3.8)		< 0.01
Waist circumference (cm)	86.7 (8.4)		89.5 (9.6)		< 0.01	81.0 (9.7)		88.0 (10.0)		< 0.01
Visceral fat area (cm^2^)	94.5 (46.5)		112.1 (50.1)		< 0.01	60.6 (37.7)		97.8 (47.6)		< 0.01
Subcutaneous fat are (cm^2^)	122.1 (56.5)		126.0 (59.0)		0.40	143.9 (78.1)		185.9 (81.3)		< 0.01
Healthy lifestyle characteristics										
Alcohol consumption (non-daily drinker)	669 (77.1)		136 (74.7)		0.50	722 (90.0)		54 (98.2)		0.04
Smoking behavior (non-smoker)	410 (82.8)		99 (80.5)		0.54	468 (90.7)		36 (97.3)		0.17
Exercise frequency (≥ 2 times per week)	148 (30.6)		33 (28.0)		0.58	133 (28.1)		11 (31.4)		0.67
Body mass index 18.5–24.9 kg/m^2^	524 (60.4)		88 (48.4)		< 0.01	560 (69.9)		30 (54.5)		0.02
Adequate sleep duration (yes)	382 (76.9)		118 (83.1)		0.14	386 (77.8)		27 (77.1)		0.93
Breakfast (every morning)	403 (80.1)		104 (83.9)		0.34	404 (77.8)		33 (86.8)		0.19
Snack between meals (no)	341 (77.7)		81 (73.6)		0.37	331 (75.6)		24 (68.6)		0.36
Proportion of participants with 6 or 7 total number of healthy lifestyle items	116 (28.3)		29 (28.7)		0.93	149 (39.7)		12 (1.4)		0.86
Hypertension (yes)	234 (27.0)		77 (42.3)		< 0.01	147 (18.3)		20 (36.4)		< 0.01
Systolic blood pressure (mmHg)	122.3 (14.0)		126.5 (15.1)		< 0.01	117.0 (15.6)		126.2 (13.0)		< 0.01
Diastolic blood pressure (mmHg)	74.8 (10.5)		74.1 (11.4)		0.38	70.5 (10.6)		74.4 (9.5)		< 0.01
Heart rate (beats per min)	68.1 (10.1)		69.8 (11.0)		0.04	72.2 (10.8)		74.0 (13.0)		0.25
Diabetes-related items										
Fasting blood glucose (mg/dl)	98.9 (8.8)		135.3 (23.3)		< 0.01	93.9 (8.9)		130.1 (27.1)		< 0.01
Hemoglobin A1c (%)	5.7 (0.3)		7.0 (0.8)		< 0.01	5.7 (0.3)		6.8 (0.8)		< 0.01
Immuno-reactive insulin	8.4 (5.5)		10.0 (5.7)		< 0.01	7.2 (5.3)		11.3 (6.9)		< 0.01
C-peptide	1.9 (0.9)		2.1 (0.6)		0.21	1.5 (0.6)		2.3 (0.7)		< 0.01
Homeostasis model assessment of insulin resistance	2.1 (1.5)		3.4 (2.1)		< 0.01	1.7 (1.3)		3.5 (2.1)		< 0.01
Dyslipidemia (yes)	369 (42.5)		90 (49.5)		0.09	279 (348)		25 (5.5)		0.11
Total cholesterol (mg/dl)	201.2 (32.4)		190.4 (36.1)		< 0.01	215.3 (33.4)		202.5 (37.8)		< 0.01
High-density-lipoprotein cholesterol (mg/dl)	56.2 (14.6)		52.2 (12.0)		< 0.01	68.5 (15.4)		59.2 (14.6)		< 0.01
Low-density-lipoprotein cholesterol (mg/dl)	114.4 (28.5)		106.4 (29.4)		< 0.01	118.8 (28.8)		112.3 (27.3)		0.11
Triglycerides (mg/dl)	124.1 (82.8)		140.8 (91.3)		0.02	90.0 (49.3)		121.4 (87.0)		< 0.01
Hyperuricemia (yes)	224 (25.8)		42 (23.2)		0.46	17 (2.1)		7 (12.7)		< 0.01
Uric acid (mg/dl)	6.1 (1.2)		5.9 (1.2)		0.01	4.7 (1.1)		5.4 (1.3)		< 0.01
High-sensitivity C-reactive protein (mg/dl)	0.12 (0.28)		0.14 (0.26)		0.21	0.10 (0.39)		0.21 (0.50)		0.06
Organ damage/cardiovascular disease										
Heart	29 (3.3)		14 (7.7)		< 0.01	17 (2.1)		3 (5.5)		0.11
Brain	17 (2.0)		1 (0.5)		0.34	16 (2.0)		0 (0.0)		0.62

The proportions of hypertension and SBP were significantly higher in DM compared to non-DM participants in both sexes. Mean HDL-C was significantly lower and TGs higher in DM compared to non-DM participants among both sexes.

Table 2  shows the results of the multivariable logistic regression analysis among men. Q4 (≥ 125 cm^2^) of VFA was significantly positively associated with DM compared to Q1 (Q4, AOR = 1.94, 95% CI 1.02–3.71). There was no association between SFA and DM. Among women, Q4 (≥ 85 cm^2^) of VFA was significantly positively associated with DM compared to Q1 (Q4, AOR = 6.15, 95% CI 1.65–22.99) (Table 3). In addition, Q3 and Q4 of SFA were significantly positively associated with DM compared to Q1 (Q3, AOR = 5.64, 95% CI 1.21–26.25; Q4, AOR = 7.81, 95% CI 1.71–35.65).

**Table 2:** Odds ratios and 95% confidence intervals for diabetes mellitus among men (logistic regression analysis) (n = 1050)

	Number	Univariate			Multivariable		
		OR	95% CI	*P*	AOR	95% CI	*P*
Visceral fat area (cm^2^)							
Q1 < 65	252	Ref			Ref		
65 ≤ Q2 < 95	285	1.26	0.75–2.09	0.38	1.13	0.60–2.13	0.71
95 ≤ Q3 < 125	236	1.86	1.13–3.08	0.02	1.67	0.88–3.16	0.11
125 ≤ Q4	274	2.44	1.52–3.93	< 0.01	1.94	1.02–3.71	0.04
Subcutaneous fat area (cm^2^)							
Q1 < 85	282	Ref			Ref		
85 ≤ Q2 < 115	236	0.87	0.55–1.38	0.55	0.69	0.37–1.29	0.24
115 ≤ Q3 < 155	270	0.83	0.53–1.31	0.43	0.97	0.54–1.73	0.91
155 ≤ Q4	262	1.10	0.71–1.68	0.68	1.16	0.83–2.58	0.19

OR: odds ratio; AOR: adjusted odds ratio; CI: confidence interval

Multivariable analysis was adjusted for visceral/subcutaneous fat area quartiles, age (years), dyslipidemia (yes), hypertension (yes), hyperuricemia (yes), alcohol consumption (non-daily drinker), and smoking (non-smoker)

**Table 3:** Odds ratios and 95% confidence intervals for diabetes mellitus among women (logistic regression analysis) (n = 857)

	Number	Univariate			Multivariable		
		OR	95% CI	*P*	AOR	95% CI	*P*
Visceral fat area (cm^2^)							
Q1 < 30	205	Ref			Ref		
30 ≤ Q2 < 60	237	1.31	0.36–4.69	0.68	1.05	0.23–4.87	0.95
60 ≤ Q3 < 85	192	3.35	1.06–10.57	0.04	2.35	0.56–9.84	0.24
85 ≤ Q4	219	8.92	3.01–25.65	< 0.01	6.15	1.65–22.99	< 0.01
Subcutaneous fat area (cm^2^)							
Q1 < 90	219	Ref			Ref		
90 ≤ Q2 < 135	195	1.52	0.52–4.46	0.45	2.23	0.40-12.54	0.36
135 ≤ Q3 < 190	222	2.76	1.06–7.18	0.04	5.64	1.21–26.25	0.03
190 ≤ Q4	221	4.53	1.82–11.27	< 0.01	7.81	1.71–35.65	< 0.01

OR: odds ratio; AOR: adjusted odds ratio; CI: confidence interval

Multivariable analysis was adjusted for visceral/subcutaneous fat area quartiles, age (years), dyslipidemia (yes), hypertension (yes), hyperuricemia (yes), alcohol consumption (non-daily drinker), and smoking (non-smoker)

**Fig. 1:**
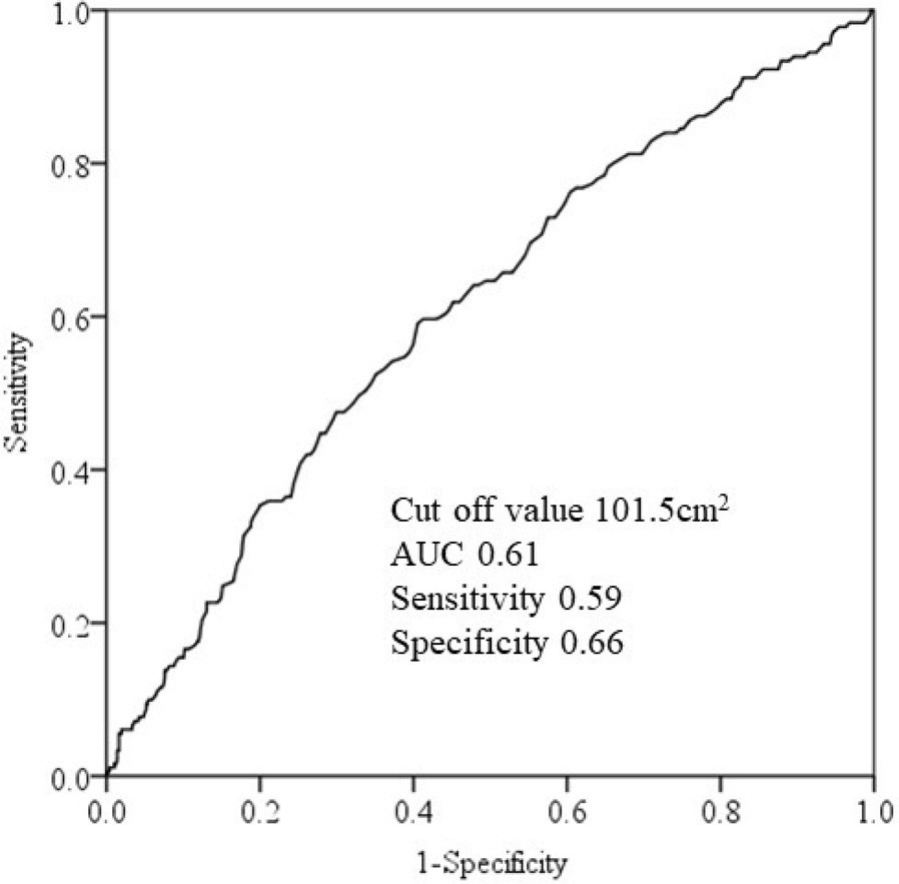
Analysis of visceral fat area receiver operating characteristic curve for diabetes mellitus in men. AUC: area under the curve

**Fig. 2:**
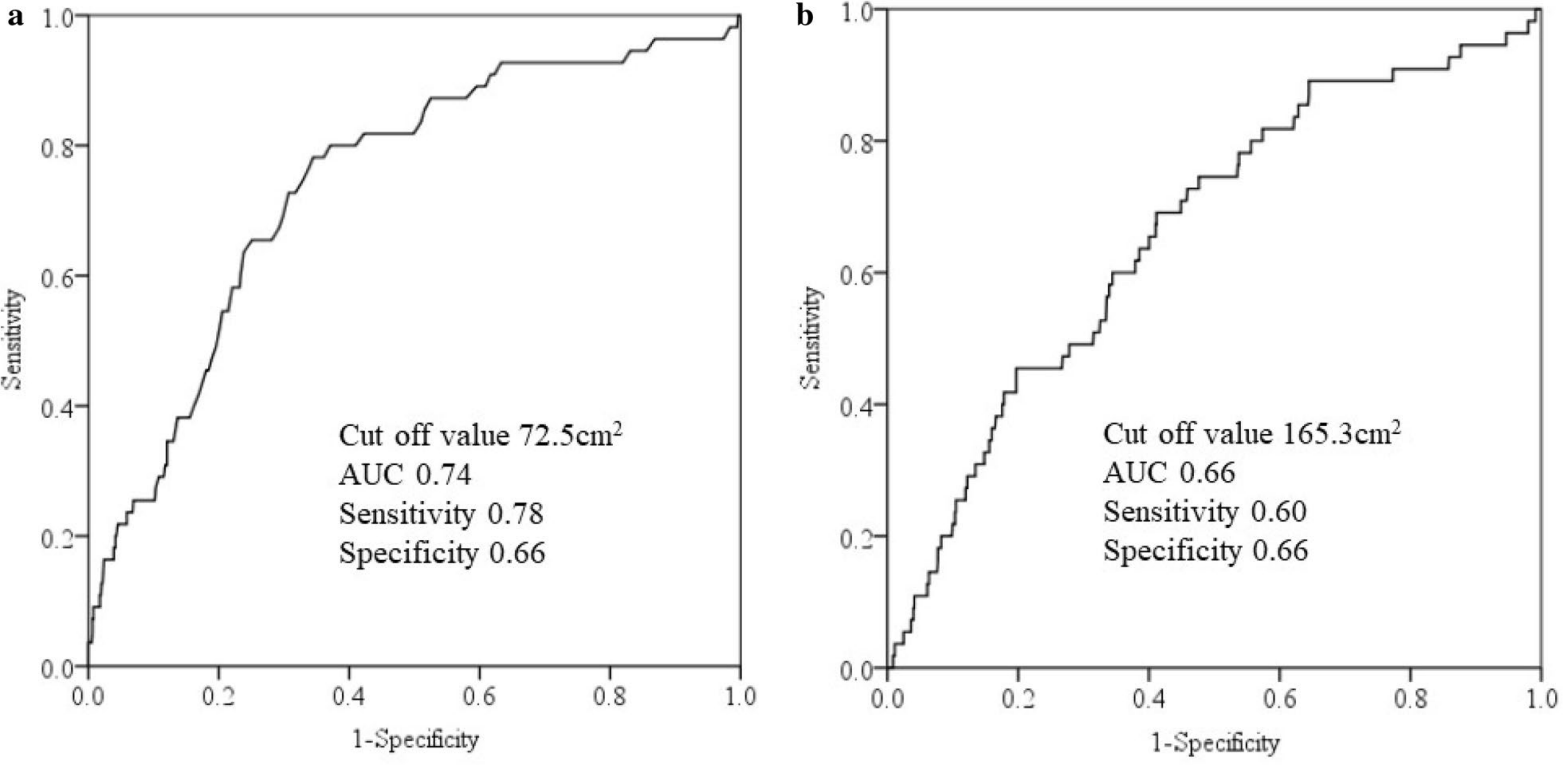
Analysis of visceral fat area (**a**) and subcutaneous fat area (**b**) receiver operating characteristic curves for diabetes mellitus in women. AUC: area under the curve

The appropriate VFA cut-off value, sensitivity, specificity, and AUC in men were 101.5 cm^2^, 0.61, 0.59, and 0.66, respectively (Fig. 1) and 72.5 cm^2^, 0.74, 0.78, and 0.66, respectively, in women (Fig. 2a). The appropriate SFA cut-off value, sensitivity, specificity, and AUC in women were 165.3 cm^2^, 0.66, 0.60, and 0.66, respectively (Fig. 2b).

## Discussion

The present cross-sectional study results showed that VFA ≥ 125 cm^2^ was significantly positively associated with DM compared to VFA < 65 cm^2^ in men, and VFA ≥ 85 cm^2^ was significantly positively associated with DM compared to < 30 cm^2^ in women after adjusting for confounders. SFA ≥ 135 cm^2^was significantly positively associated with DM compared to SFA < 90 cm^2^ in women, whereas no association was observed in men. VFA was closely and positively associated with DM in both sexes, and appropriate estimated cut-off points might be 101.5 cm^2^ in men and 72.5 cm^2^ in women for DM, respectively. SFA was also associated with DM only in women, suggesting a cut-off value of 165.3 cm^2^. To the best of our knowledge, analyses of the association between DM and VFA and SFA are limited.

Visceral fat accumulation is widely regarded as a risk factor for cardiovascular diseases, including DM. Mets is a metabolic condition that predicts individuals who are likely to be affected by cardiovascular disorders via insulin resistance [3]. One major feature of Mets is visceral fat accumulation, which is closely related to insulin resistance. Visceral fat accumulation is generally recognized as a WC ≥ 85 cm^2^ in men and ≥ 90 cm^2^ in women, which correspond to a VFA of 100 cm^2^ in an abdominal CT scan at the umbilical level [3]. Visceral fat accumulation is also known to be an independent risk factor for type 2 diabetes. A longitudinal study that determined the optimal cut-off value of VFA for predicting type 2 diabetes among 13,004 Koreans reported values of 118.8 cm^2^ in men and 82.6 cm^2^ in women [4]. Another longitudinal survey that followed Japanese Americans for 10 years reported a baseline intra-abdominal fat area (IFA) of 102.7 cm^2^ in the incident diabetes group and 74.3 cm^2^ in those without incident diabetes, respectively. Also, an increase of 1 SD in IFA was associated with a 1.65-fold increase in the odds of diabetes over 10 years (OR = 1.65, 95% CI 1.21–2.25) after adjusting for the above covariates [19]. These previous study results are closely similar to our results. Thus, sex-specific reference values for visceral fat accumulation such as that men with a VFA ≥ 100 cm^2^ and women with a VFA ≥ 80 cm^2^ should be considered to prevent and manage type 2 diabetes.

Our results showed that SFA was significantly positively associated with DM in women, whereas no association was observed in men. The role of subcutaneous fat in cardiovascular risk remains controversial. The Shanghai Nicheng Cohort Study, which was conducted among 12,137 Chinese adults aged 45–70 years, reported multivariable-adjusted ORs and 95% CIs of newly diagnosed diabetes per 1-standard deviation increase in SFA and VFA of 1.29 (1.19–1.39) and 1.61 (1.49–1.74) in men and 1.10 (1.03–1.18) and 1.56 (1.45–1.67) in women, respectively [6]. However, the association between SFA and newly diagnosed diabetes disappeared in men and was reversed in women (OR 0.86 [95% CI 0.78–0.94]) after additional adjustment for BMI and VFA [6]. A study that surveyed 3001 participants from the Framingham Heart Study reported that multivariable-adjusted general linear regression analyses of SAT and VAT showed significant associations with blood glucose in both sexes [age-adjusted Pearson correlation coefficients; 0.23 for SAT and 0.34 for VAT in women (P < 0.001), 0.12 for SAT and 0.19 for VAT in men (P < 0.001)]. In addition, the magnitude of association between VAT and all risk factors was greater for women than men, and weaker sex differences were observed for SAT [8]. The Jackson Heart Study, which surveyed 2477 African Americans, reported that abdominal VAT and SAT were both associated with adverse cardiometabolic risk factors, including diabetes, and the effect size of VAT in women was larger than that of SAT [fasting plasma glucose, 5.51 ± 1.0 vs. 3.36 ± 0.9; diabetes, 1.82 (1.6–2.1) vs. 1.58 (1.4–1.8); and Mets, 3.34 (2.8–4.0) vs. 2.06 (1.8–2.4), respectively; P < 0.0001 for difference between VAT and SAT] [9]. The possible mechanism of the association between diabetes and SAT as well as VAT is insulin resistance. To date, numerous studies have assessed the association between excess visceral fat accumulation and insulin resistance. Regarding SAT, several previous surveys indicated a positive association between excess subcutaneous fat accumulation and insulin resistance. A Japanese study that surveyed 912 non-diabetic participants reported that subjects in higher tertiles of SAT and VAT had significantly higher HOMA-IR and lower Matsuda ISI levels (P < 0.001) [20]. Excess SAT accumulation may cause insulin resistance and contribute to glucose intolerance as well as VAT. Therefore, it is necessary to consider adiposity, including SAT and VAT, to better maintain body composition.

In regard to the impact of SAT, a sex difference was observed. There is little evidence available to explain the difference. The Jackson Heart Study, which involved 2,799 African Americans, reported a direct association between SAT and adiponectin (β = 0.18; P = 0.002) that persisted when controlling for BMI and WC among men, whereas the significance was borderline among women (β = 0.05; P = 0.05) [21]. Although the evidence is limited to explain the sex difference, it is possible that adiponectin may contribute. Further analyses are required to assess the sex difference.

Our study has several limitations. First, it was susceptible to selection bias, as the participants consisted of those who received voluntary medical check-ups at a single medical institution. As such, these participants may be inherently more aware of their health behaviors relative to the general population. In addition, 978 among 2885 screened participants were excluded due to missing data (33.9%). It is necessity to minimize the exclusion rate. Second, this was a cross-sectional observational study, thus limiting consideration of the causal relationship between SFA/VFA and DM. Further analyses that include data from a more diverse cohort are thus needed. Third, some key data regarding items such as details of diabetes medications, eating behaviors, and nutritional status were not collected. Future prospective studies including these data are also needed.

In conclusion, the results of the present cross-sectional study indicate that VFA ≥ 125 cm^2^ in men is significantly positively associated with DM compared to VFA < 65 cm^2^, and VFA ≥ 85 cm^2^ in women is significantly positively associated with DM compared to VFA < 30 cm^2^ after adjusting for confounders. SFA ≥ 135 cm^2^ in women is significantly positively associated with DM compared to SFA < 90 cm^2^, but no association was observed in men. Appropriate estimated VFA cut-off points for DM are 101.5 cm^2^ in men and 72.5 cm^2^ in women, respectively. As SFA was associated with DM only in women, the appropriate estimated cut-off is 165.3 cm^2^. Our results suggest that it is important to consider both SFA and VFA, especially in women, for primary and secondary prevention of DM.

## Data Availability

The ethics committee imposed restrictions to data access and sharing. Individuals who wish to access our data must obtain further permission from the committee, which can be achieved by contacting the corresponding author.
